# Histopathology of Corn Plants Infected by Endophytic Fungi

**DOI:** 10.3390/biology11050641

**Published:** 2022-04-22

**Authors:** Tersoo P. Terna, Nik Mohd Izham Mohamed Nor, Latiffah Zakaria

**Affiliations:** 1 Department of Plant Science and Biotechnology, Federal University of Lafia, PMB 146, Lafia 950101, Nigeria; ternapaul@yahoo.com; 2 School of Biological Sciences, Universiti Sains Malaysia, Penang 11800, Malaysia; nikizham@usm.my

**Keywords:** endophytic fungi, colonization, corn tissues, disease response, light microscopy, transmission electron microscopy

## Abstract

**Simple Summary:**

Endophytic fungi are fungi that live all or part of their life cycles within the tissues of their hosts. Although several endophyte-plant interactions are non-pathogenic in nature, it has been reported that upon infection, some endophytic fungi are able to adopt a pathogenic status resulting in disease symptoms in their host plants, ranging in severity from mild to severe. In the present study, light microscopy and transmission electron microscopy were used to investigate the tissue colonization and cell damage of corn plants by three endophytic fungi, *Fusarium verticillioides, Fusarium sacchari,* and *Penicillium citrinum,* causing disease symptoms in the roots, stems, and leaves of infected corn plants. Tissue deterioration and cell collapse were produced by endophytic *F. verticillioides,* while significant cell proliferation and wall thickening were observed in corn plants infected by endophytic *F. sacchari.* Corn plants infected by *P. citrinum* showed reductions in cell diameter of the vascular bundles of infected corn tissues. The ability of endophytic fungi recovered from healthy corn plants to cause disease when inoculated in healthy tissues of other corn plants signifies their importance as determinants of corn health and productivity.

**Abstract:**

Endophytic fungi inhabiting plant tissues show extensive functional diversity, ranging from mutualism to pathogenicity. The present study evaluated the histological responses of corn plants to colonization by three species of endophytic fungi isolated from corn. Corn seedlings were inoculated with 1 × 10^6^ conidia per mL spore suspensions of endophytic *Fusarium verticillioides, Fusarium sacchari,* and *Penicillium citrinum* and observed for 14 days for the emergence of disease symptoms. Histological examination of diseased root, stem, and leaf tissues was conducted using light and transmission electron microscopy. The results indicated that the mean diameters of root phloem, stem vascular bundles, and leaf vascular bundles, of corn plants infected with endophytic *P. citrinum* (18.91 µm, 146.96 µm, and 107.86 µm, respectively), *F. verticillioides* (18.75 µm, 85.45 µm, and 118.24 µm, respectively), and *F. sacchari* (24.15 µm root phloem, and 98.90 µm stem vascular bundle diameters), were significantly lower than the root phloem (33.68 µm), stem vascular bundle (186.77 µm), and leaf vascular bundle (155.88 µm) of the uninfected corn plants (*p* ≤ 0.05). Endophytic *F. verticillioides* was the most virulent, resulting in severe degradation and the eventual collapse of infected plant tissues. The study showed that endophytic fungi recovered from corn plants are capable of initiating significant disease responses in infected corn tissues.

## 1. Introduction

Endophytes are microbes that live all or part of their life cycles in the intercellular and intracellular spaces of living and apparently healthy host plant tissues, while the inhabited host tissues remain intact and functional most of the time [1]. Fungal endophytes form a diverse group of microorganisms living within the roots and/or shoots of the plant, and most plants in natural ecosystems appear to host fungal endophytes. This highly diverse group of fungi can have profound impacts on plant communities through increasing fitness by conferring abiotic and biotic stress tolerance, and increasing biomass. They can also decrease fitness by altering resource allocation [2].

In corn, endophytic fungi belonging to the genera *Fusarium* and *Penicillium* have been recovered from different plant parts such as roots, stems, leaves, and kernels [3,4,5]. Although the infection of corn tissues by fungal endophytes belonging to the genus *Fusarium* has been linked with various disease symptoms such as a decrease in shoot diameter, plant height, leaf length, chlorophyll content, and plant dry weight, accompanied by various histological responses such as the collapse of infected cells [3,6,7], much remains unknown regarding the pathogenicity and cellular responses of corn plants infected by endophytic *Penicillium* spp. In the present study, the tissue colonization and histopathology of corn tissues infected with endophytic fungi, *Fusarium verticillioides, Fusarium sacchari,* and *Penicillium citrinum* were evaluated. Histological techniques using light and electron microscopy play important roles in the structural description of the infection process and in host responses in several endophyte-host plant associations [8]. A combination of light and electron microscopy is also able to reveal the precise internal locations of endophytic fungi; hence, microscopic evidence to confirm the location of the fungus is recommended for a much more conclusive designation of the endophytic status of the fungus [9]. These techniques are generally targeted at establishing the colonization pattern (i.e., intercellular or intracellular) and examining the cellular anatomy of host plants [10].

Although studies involving the visualization of endophytic fungi have been carried out in several tropical grasses and the Pinaceae using fresh tissues or electron microscopy techniques [11,12], few researchers have reported the use of histological techniques to demonstrate endophytic colonization of corn host tissues. For example, Yates et al. [3] reported the alteration of gross morphology and the histology of corn seedlings during symptomless endophytic infection by *F. verticillioides.* Bacon and Hinton [13] reported the occurrence of intercellular hyphae in symptomless corn plants colonized by *F. verticillioides,* which persisted throughout the plant. The present work investigated the pathogenic interactions of endophytic fungi in the root, stem, and leaf tissues of corn plants showing disease symptoms after infection with endophytic *F. verticillioides, F. sacchari,* and *P. citrinum,* using light and transmission electron microscopy.

## 2. Materials and Methods

### 2.1. Source of Endophytic Fungi

Endophytic *F. verticillioides*, *F. sacchari*, and *P. citrinum* isolates used in the study were recovered from healthy tissues of sweet corn, of which *F. verticillioides* and *P. citrinum* were recovered from corn kernels, while *F. sacchari* was isolated from husks of corn plants. The recovery of endophytic fungi from corn tissues was carried out on sterile potato dextrose agar (PDA) after surface sterilization of disease-free tissues in 5% sodium hypochlorite solution, followed by 70% ethanol for 2 min each. The efficacy of surface sterilization was confirmed using the imprint technique [14]. The three endophytic fungi were selected for histopathology studies after producing disease symptoms such as stunted growth, stem rot, leaf necrosis, leaf chlorosis, and plant wilt, in artificially inoculated sweet corn plants.

Endophytic fungi were identified through molecular identification using two molecular markers for each species. For molecular identification of *Fusarium,* β-tubulin and TEF-1α genes were applied, using the primer pairs T1 and T2 [15], for amplification of β-tubulin and EF1 and EF2 [16] for amplification of TEF-1α. For *Penicillium*, the ITS region and β-tubulin gene were applied, using the primer pairs ITS1 and ITS4 [17], for amplification of the ITS region and Bt2a and Bt2b [18] for the amplification of the β-tubulin gene. The PCR reaction mixture was prepared in 50 μL total volume comprising 5× Green Buffer (8 μL), MgCl_2_ (8 μL), 1.0 μL of dNTP mix (Promega, Madison, WI, USA), 8 μL of each primer (5.0 μM for ITS and TEF-1α, 2.5 μM for β-tubulin), 0.3 μL of 5U/μM GoTaq^®^ DNA polymerase (Promega, USA), and 0.6 μL of DNA template. The reaction mixture was brought to a total volume of 50 μL with sterile distilled water.

PCR amplifications were performed in a Bio-Rad MyCycler™ Thermal Cycler. PCR amplifications for ITS commenced with an initial denaturation at 95 °C for 5 min, followed by 35 cycles of denaturation at 95 °C for 30 s, annealing at 56 °C for 30 s, and extension at 72 °C for 1 min. Final extension was performed at 72 °C for 5 min. For TEF-1α, initial denaturation was performed at 94 °C for 2 min, followed by 35 cycles of denaturation at 94 °C for 30 s, annealing at 59 °C for 30 s, and extension at 72 °C for 1 min. The final extension of the TEF-1α gene was carried out at 72 °C for 10 min. PCR reactions for β-tubulin commenced with an initial denaturation at 94 °C for 1 min, followed by 39 cycles of denaturation at 94 °C for 30 s, annealing at 58 °C for 30 s, and extension at 72 °C for 1 min. Final extension of β-tubulin gene was performed at 72 °C for 5 min. PCR products were detected by electrophoresis in 1% agarose gel prepared in 1× Tris Borate-EDTA (TBE) buffer, stained with 4 µL of Florosafe DNA Stain (1st Base, Singapore). PCR products were sent for sequencing to a service provider.

### 2.2. Seed Inoculation and Corn Growth

Seeds of sweet corn, Leckat 592 variety, were purchased from the Leckat Corporation, Kuala Lumpur, Malaysia. Corn seeds were subjected to external and internal sterilization, using the heat shock procedure reported by Palencia [19], and pre-germinated until the emergence of radicle and plumule by plating them with embryos facing downwards on sterile water agar for three days [20]. Pre-germinated corn seedlings at the rate of 10 seedlings per plate were inoculated by overnight incubation in 10 mL conidial suspensions (1 × 10^6^ conidia per mL of distilled water) of endophytic fungi, at room temperature, in a laminar flow chamber.

Pre-germinated corn seedlings inoculated with fungal spores were aseptically transferred into sterile sandy-clay soils in sterile plastic containers, measuring 17 × 7 cm (width × height). Planting depth was standardized by placing each seed in a 2.5 cm deep depression in the growth soil prior to covering the seed with soil [3]. The experiment was laid out using the completely randomized design with four treatment replicates. Each treatment comprised a single fungal isolate inoculated on a pre-germinated corn seedling, while the control group consisted of seedlings treated with sterilized distilled water without a fungal inoculum. All plants were grown for 14 d in the Plant House, School of Biological Sciences, Universiti Sains Malaysia, Penang and watered every 24 h prior to shoot emergence, and twice daily (morning and evening) after emergence.

Roots, stems, and leaves of 14-day-old infected plants showing disease symptoms such as poor root formation, plant wilt, stunted growth, stem rot, stem malformation, leaf necrosis, and chlorosis were collected for histological studies. Root tissues about 2 cm in length were excised 1 cm away from the tip of adventitious roots, while stem tissues about 2 cm long were cut from stem portions between the first node and first collar of infected stems. Leaf tissues approximately 2 × 2 cm (length × width) including the mid-rib were excised at diseased portions for subsequent processing and microscopy. Sampled tissues were surface sterilized in 5% sodium hypochlorite solution for 2 min, followed by 70% ethanol for 2 min. After surface sterilization, sampled plant tissues were plated on PDA for three days to confirm the presence of the inoculated endophytic fungi before it was fixed in formalin acetic acid (FAA).

### 2.3. Sample Preparation for Light Microscopy

Dehydration, embedding, sectioning, and staining of diseased tissues were carried out in accordance with the procedures of Livingston et al. [21]. Prepared slides of endophyte-infected tissues of corn plants were viewed, and photomicrographs were taken at magnifications 40×, 200×, and 400× using a photomicrograph-enabled light microscope (LM) (Olympus BX41). Measurements of histological features of infected and uninfected root, stem, and leaf samples of corn plants were taken from four different sections of each plant part, using the Cell A Olympus camera software. The thickness of the epidermis; the cortical parenchyma diameter; the thickness of endodermis; the pericycle diameter; the protoxylem diameter; the phloem diameter; the metaxylem diameter; the diameter of the sclerenchyma cells; the bundle sheath diameter; and the vascular bundle diameter of infected tissues were measured. Cells of infected corn tissues were also observed for the presence of endophytes.

### 2.4. Transmission Electron Microscopy

Endophyte-infected plant tissues preserved in McDowell-Trump fixative were processed, sectioned on an ultramicrotome (PowerTome XL-RMC Products, Boeckeler Intruments Inc., Tucson, AZ, USA), and stained, using the methods of Guzmán et al. [22]. Following tissue preparation, stained ultrasections of endophyte-infected corn tissues were viewed and photomicrographs taken under a transmission electron microscope (TEM) (Zeiss EFTEM Libra 120, Jena, Germany).

### 2.5. Data Analysis

Measurements of histological responses of endophyte-infected corn plants were subjected to Analysis of Variance, using IBM SPSS statistical software version 27 (Chicago, IL, USA). Means were separated using the Tukey’s Honestly Significant Difference test at 5% level of probability.

## 3. Results

### 3.1. Endophytic P. citrinum, F. verticillioides, and F. sacchari in Corn Tissues

Endophytic *P. citrinum*, *F. verticillioides*, and *F. sacchari* showed variations in their occurrence in the sampled corn tissues (Table 1). Both *F. verticillioides* and *F. sacchari* showed 8.57% occurrence in husk tissues, while the occurrence of *P. citrinum* in husk tissues was 1.40%. In corn kernels, *P. citrinum* showed the highest occurrence (28.20%) compared to *F. verticillioides* (3.93%), while *F. sacchari* was absent.

### 3.2. Pathogenicity of Endophytic Fungi

Disease symptoms initiated by endophytic fungi in infected plant tissues are presented in Table 2. All three endophytic fungi induced a reduction in root formation and stem narrowing, while reduced stem elongation and stem rot were observed in *F. verticillioides*-infected corn plants. Stem infection by endophytic *F. sacchari* resulted in excessive stem elongation and stem malformation, while the fungus was absent in the leaves of infected plants. Leaves infected by endophytic *F. verticillioides* and *P. citrinum* were both necrotic and chlorotic.

### 3.3. Histology of Uninfected Corn Roots

**Figure 1:**
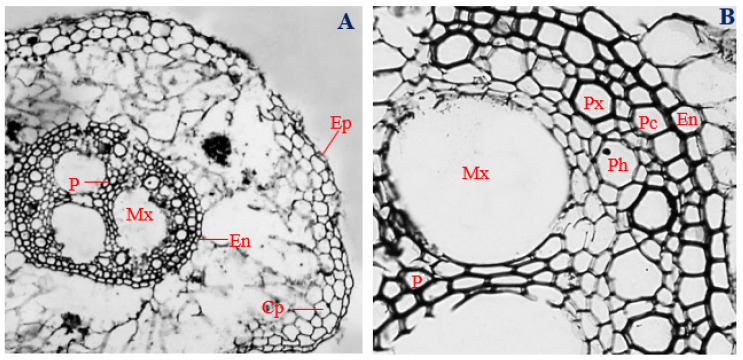
The transverse section of the uninoculated corn root. (**A**) Magnification 40×; (**B**) magnification 200×. Ep, epidermis; Cp, cortical parenchyma; En, Endodermis; Pc, pericycle; Px, protoxylem; Ph, phloem; Mx metaxylem; and P, pith.

Histological characteristics of uninfected roots (control) of corn plants are presented in Figure 1. Epidermal cells of uninfected corn roots were horizontally elongated, uniseriate, overlapping, varying in shape from spindly to roughly circular, and irregular, with a mean thickness of 14.69 µm (Figure 1A,B). Cortical parenchyma cells ranged in shape from isodiametric to irregular, with a mean diameter of 34.94 µm, and distributed mostly at the outer regions of the cortex (Figure 1A,B).

In control corn plants, the cells of the endodermal layer were horizontally elongated, ranging in shape from rectangular to roughly circular and rarely showing a “U-shaped” thickening, with a mean thickness of 17.43 µm (Figure 1A,B). The pericycles of uninfected roots of corn plants were double-layered, having overlapping cells that were mostly polygonal in shape and elongated horizontally, with a mean diameter of 19.46 µm (Figure 1A,B).

Protoxylem vessels, ranging in shape from polygonal to roughly circular, with a mean diameter of 34.71 µm, surrounded the pith and metaxylem vessels and were arranged interchangeably with polygonal-shaped phloem cells, having a mean diameter of 33.68 µm (Figure 1A,B). Three roughly circular metaxylem vessels measuring about 129.49 µm mean diameter surrounded the central pith. A narrow but clearly defined pith, made up of parenchyma cells with different shapes, ranging from polygonal, to spindle-shaped, to rectangular, to circular, and to irregular, was observed at the central position of the vascular cylinder (Figure 1A,B). The vascular bundle of uninfected root sections of corn plants had a mean diameter of 396.31 µm.

### 3.4. Histology of Corn Roots Infected with Endophytic F. verticillioides

**Figure 2:**
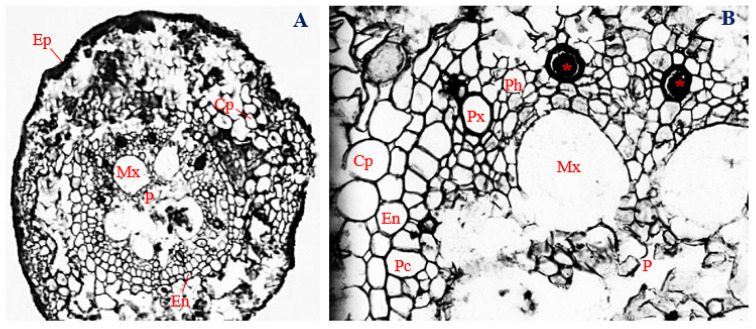
The transverse section of corn roots infected with endophytic *Fusarium verticillioides.* (**A**) Magnification 40×; (**B**) magnification 200×. Ep, epidermis; Cp, cortical parenchyma; En, Endodermis; Pc, pericycle; Px, protoxylem; Ph, phloem; Mx, metaxylem; and P, pith. Occluded xylem vessels are marked with asterisks (*).

In the roots of corn plants infected with endophytic *F. verticillioides*, cells of the epidermal layer collapsed and were almost unrecognizable (Figure 2A). The epidermal layer was uniseriate and had a mean thickness of 14.39 µm. In the cortex, the cells of the cortical parenchyma ranged in shape from isodiametric to irregular, with a mean diameter of 30.33 µm.

Unlike the uninfected control, cells of the cortical parenchyma of *F. verticillioides*-infected corn roots were mostly localized within the cortex and enveloping the endodermis. Towards the center of the root section, an endodermal layer, about 16.45 µm thick, was observed. Cells of the endodermal layer were mostly polygonal in shape, with a few showing “U-shaped” thickening (Figure 2B).

Below the endodermis, a vascular cylinder, with a mean diameter of 401.22 µm, was encased by a single-layered pericycle, comprising overlapping and vertically elongated cells, ranging in shape from polygonal, to roughly circular, to rectangular, and to irregular. Cells of the pericycle layer had a mean diameter of 19.55 µm. Immediately below the pericycle, polygonal-shaped protoxylem vessels about 27.50 µm in diameter were arranged interchangeably with roughly circular to oval shaped phloem vessels, having a mean diameter of 18.75 µm (Figure 2B).

Five roughly circular to irregular-shaped metaxylem vessels, about 95.51 µm in diameter, surrounded the pith region, towards the center of the vascular cylinder. At the center of the vascular cylinder, a severely degraded and collapsed pith was observed. Cells of the pith region ranged in shape from roughly circular to irregular (Figure 2A,B). The occlusion of protoxylem vessels was also evident (Figure 2B).

Cells of the vascular cylinder were much reduced in size in *F. verticillioides*-infected roots (Figure 2A,B), compared to the control (Figure 1A,B). Differences in the mean sizes of conducting tissues (phloem and metaxylem) between the roots of corn plants infected with endophytic *F. verticillioides* and the uninfected control were significant (*p* ≤ 0.05) (Figure 1B and Figure 2B).

### 3.5. Histology of Corn Roots Infected with Endophytic F. sacchari

**Figure 3:**
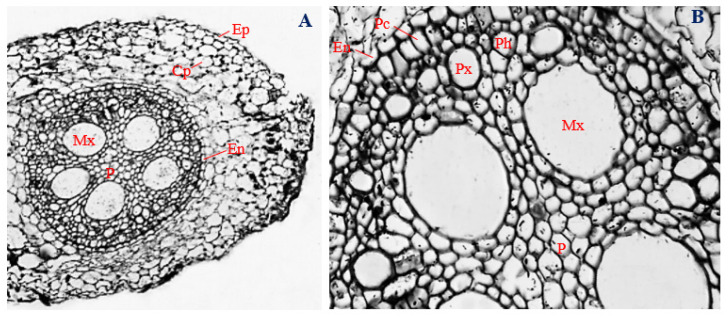
The transverse section of corn roots infected with endophytic *Fusarium sacchari.* (**A**) Magnification 40×; (**B**) magnification 200×. Ep, epidermis; Cp, cortical parenchyma; En, Endodermis; Pc, pericycle; Px, protoxylem; Ph, phloem; Mx, metaxylem; and P, pith.

The epidermal layers of corn roots infected with endophytic *F. sacchari* were uniseriate and consisted of oval to irregular-shaped cells, elongated horizontally and curved inwards, with a mean diameter of 23.73 µm (Figure 3A,B). Cortical parenchyma cells of *F. sacchari*-infected corn roots were roughly circular to irregular in shape, with a mean diameter of 31.73 µm. However, unlike the uninfected roots, the cortical parenchyma of *F. sacchari*-infected roots was densely packed and distributed throughout the cortex. The endodermal layer had a mean thickness of 13.47 µm and was mostly comprised of “U-shaped” cells that were elongated longitudinally (Figure 3A,B).

The vascular bundle was encased by a double-layered pericycle, made of vertically elongated polygonal cells (Figure 3B). Cells of the pericycle overlapped, with a mean diameter of 15.30 µm. Next to the pericycle, in the outer position of the vascular cylinder, polygonal protoxylem cells, about 30.93 µm in diameter, were arranged alternately with phloem vessels ranging in shape from polygonal to roughly circular and with a mean diameter of 24.15 µm. Around the center of the vascular cylinder, five oval-shaped metaxylem vessels, about 94.95 µm in diameter, surrounded a well-defined pith. Pith cells, mostly oval in shape, were densely packet at the central core of the vascular cylinder (Figure 3A,B). The vascular bundle of *F. sacchari*-infected roots of corn plants had a mean diameter of 418.76 µm.

Although the epidermal layers of *F. sacchari*-infected roots of corn plants (Figure 3A) were significantly thicker than the root epidermis of uninfected corn plants (Figure 1A) (*p* ≤ 0.05), the thickness of the endodermal layer, and the cell diameters of the phloem and metaxylem of corn plants infected with endophytic *F. sacchari* (Figure 3B)*,* were significantly smaller in size than those of the uninfected control (Figure 1B) (*p* ≤ 0.05).

### 3.6. Histology of Corn Roots Infected with Endophytic P. citrinum

**Figure 4:**
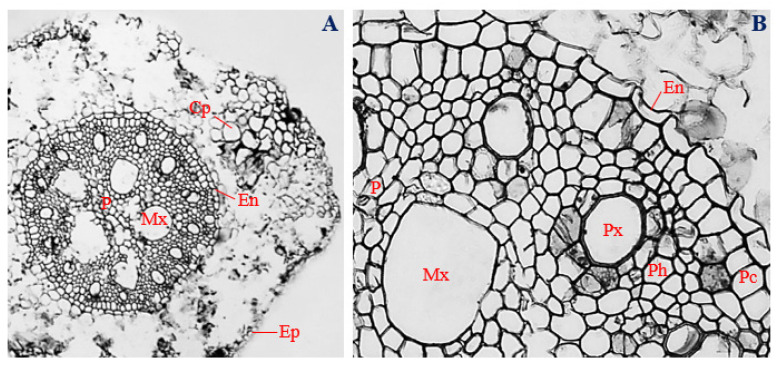
The transverse section of corn roots infected with endophytic *Penicillium citrinum*. (**A**) Magnification 40×; (**B**) magnification 200×. Ep, epidermis; Cp, cortical parenchyma; En, Endodermis; Pc, pericycle; Px, protoxylem; Ph, phloem; Mx, metaxylem; and P, pith.

Cross-sections of corn roots infected with endophytic *P. citrinum* showed the presence of uniseriate epidermis, with a mean thickness of 17.53 µm, and comprising overlapping cells of different shapes, ranging from irregular to oval (Figure 4A,B).

In the cortex of *P. citrinum*-infected roots, the cortical parenchyma consisted of various shapes, ranging from isodiametric, to oval, and to irregular, with a mean diameter of 31.42 µm (Figure 4A). Unlike uninfected roots, cortical parenchyma of corn roots infected with endophytic *P. citrinum* were localized at certain positions within the cortex (Figure 4A,B). The endodermal layer had a mean thickness of 16.44 µm, and most endodermal cells of *P. citrinum*-infected roots showed “U-shaped” thickening (Figure 4A,B).

In the vascular cylinder, a double-layered pericycle was observed. Cells of the pericycle varied in shape from polygonal, to rectangular, and to roughly circular, with a mean diameter of 22.54 µm (Figure 4B). Pericycle cells of *P. citrinum*-infected roots were vertically elongated, as opposed to horizontal elongation observed in the pericycle cells of uninfected roots (Figure 4A,B). Polygonal to roughly circular protoxylem vessels, with a mean diameter of 41.47 µm, were arranged in an alternating pattern with polygonal-shaped phloem vessels, measuring about 18.91 µm in diameter. Roughly circular to irregular metaxylem vessels, with a mean diameter of 95.30 µm, surrounded the central pith. More metaxylem vessels were observed in *P. citrinum*-infected roots (*n* = 5) (Figure 4A) compared to roots of uninfected corn plants (*n* = 3) (Figure 1A).

At the center of the vascular cylinder of *P. citrinum*-infected roots, a well-defined pith, comprising roughly circular to irregular parenchymatous cells, was observed. The pith region was wider in *P. citrinum*-infected roots (Figure 4A) compared to the uninfected control (Figure 1A). The vascular bundles of *P. citrinum*-infected roots had a mean diameter of 396.31 µm (Figure 4A).

The sizes of phloem and metaxylem vessels were significantly larger (*p* ≤ 0.05) in uninfected roots of corn plants (Figure 1B), compared to endophytic *P. citrinum*-infected plants (Figure 4B). Corn plants infected with endophytic *P. citrinum* had significantly larger vascular bundle diameters (Figure 4A), compared to uninfected corn plants (*p* ≤ 0.05) (Figure 1A).

### 3.7. Histology of Uninfected Corn Stems

**Figure 5:**
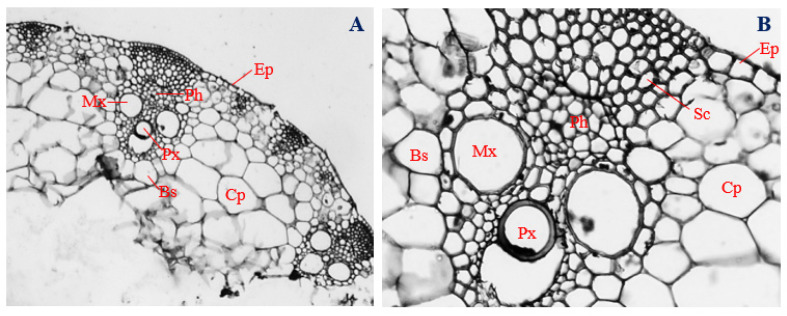
The transverse section of an uninoculated corn stem. (**A**) Magnification 200×; (**B**) magnification 400×. Ep, epidermis; Cp, cortical parenchyma; Sc, sclerenchyma; Ph, phloem; Mx, metaxylem; Px, protoxylem; and Bs, bundle sheath.

Cross-sections of the uninfected stems (control) of corn plants showed the presence of horizontally elongated epidermal cells, with a mean diameter of 27.14 µm (Figure 5A,B). Epidermal cells were arranged uniseriately and comprised different cell shapes, mostly rectangular, with a few mildly polygonal (Figure 5A,B).

Located directly below the outer epidermis, sclerenchyma cells, which were mostly polygonal in shape, had a mean diameter of 16.30 µm (Figure 5B), and polygonal-shaped bundle sheath cells, with a mean diameter of 28.04 µm, enveloped the vascular bundle (Figure 5A,B). Within the vascular bundle, two isodiametric-shaped metaxylem vessels with a mean diameter of 52.78 µm were arranged on opposite sides, while oval-shaped protoxylem vessels, measuring about 31.94 µm in diameter, were located at a central position, slightly below the mid-point between the metaxylem vessels (Figure 5A,B).

Phloem cells were positioned more externally in relation to the position of xylem cells and were mostly polygonal in shape, with a mean diameter of 12.17 µm (Figure 5A,B). Isodiametric to irregularly shaped cortical parenchyma cells, measuring about 103.36 µm, surrounded the bundle sheath and vascular bundles (Figure 5A,B). The vascular cylinder of uninfected corn stems had a mean diameter of 186.77 µm (Figure 5A).

### 3.8. Histology of Corn Stems Infected with Endophytic F. verticillioides

**Figure 6:**
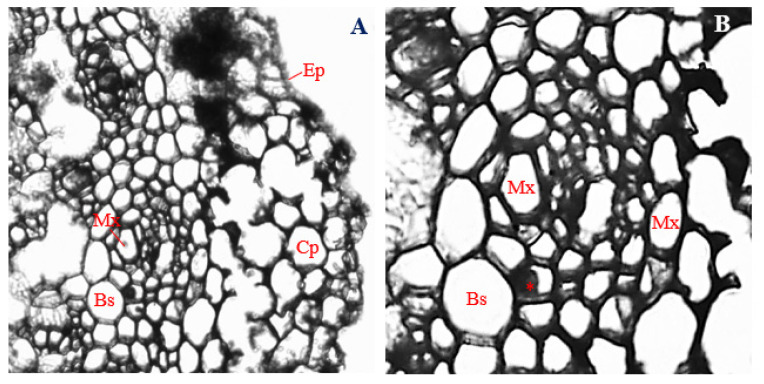
The transverse section of a corn stem infected with endophytic *Fusarium verticillioides*. (**A**) Magnification 200×; (**B**) magnification 400×. Ep, epidermis; Cp, cortical parenchyma; Mx, metaxylem; and Bs, bundle sheath. Occlusion of xylem parenchyma is marked with an asterisk (*).

Corn stems infected with endophytic *F. verticillioides* showed degradation of epidermal, cortical, and vascular tissues, such that only cells of the cortical parenchyma, bundle sheath, and metaxylem, could be easily identified (Figure 6A,B).

An epidermal layer of about 19.92 µm in thickness was observed. However, unlike stems of uninfected corn plants, layers of roughly circular to irregular overlapping parenchyma cells, about 28.04 µm in diameter, were found in-between the epidermal layer and the vascular bundles of *F. verticillioides*-infected corn stems (Figure 6B). Roughly circular to rectangular bundle sheath cells, about 25.17 µm in diameter, surrounded the vascular cylinder.

In the vascular cylinder, two recognizable metaxylem vessels, with a mean diameter of 21.20 µm and ranging in shape from polygonal to roughly circular, were observed (Figure 6B). Occlusion of xylem parenchyma was also evident (Figure 6B). Vascular bundles of corn stems infected with endophytic *F. verticillioides* had a mean diameter of 85.45 µm (Figure 6A,B). The thickness of the epidermis; the diameter of cortical parenchyma cells; metaxylem; and the vascular bundle were significantly reduced in *F. verticillioides*-infected stems of corn plants (Figure 6A,B), compared to uninfected control (Figure 5A,B) (*p* ≤ 0.05).

### 3.9. Histology of Corn Stems Infected with Endophytic F. sacchari

**Figure 7:**
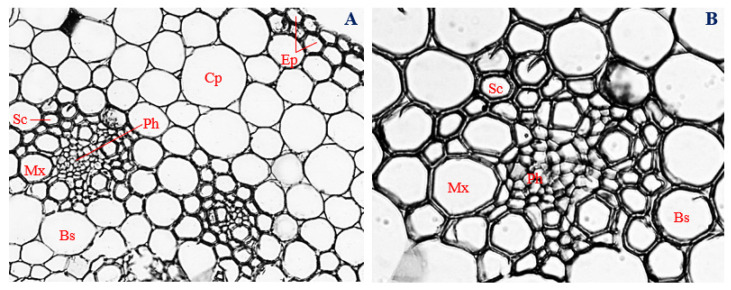
The transverse section of a corn stem infected with endophytic *Fusarium sacchari*. (**A**) Magnification 200×; (**B**) magnification 400×. Ep, epidermis; Cp, cortical parenchyma; Sc, sclerenchyma; Ph, phloem; Mx, metaxylem; and Bs, bundle sheath.

Stems of corn plants infected with endophytic *F. sacchari* showed the presence of a biseriate epidermis (Figure 7A), as opposed to the uniseriate epidermis found in uninfected corn plants (Figure 5A). The epidermal layer of *F. sacchari*-infected stems had a mean thickness of 27.83 µm and consisted of horizontally elongated polygonal to rectangular cells (Figure 7A).

Compared to stem sections of uninfected corn plants (Figure 5A), stems of *F. sacchari*-infected corn plants showed the presence of about five layers of cortical parenchyma cells in-between the epidermis and the vascular bundle (Figure 7A). Cortical parenchyma cells were mostly turgid, oval-shaped, and compactly arranged, with a mean diameter of 32.93 µm (Figure 7A).

Unlike the vascular bundles of uninfected stems (Figure 5B), vascular bundles of corn stems infected with endophytic *F. sacchari* were enclosed by large, oval-shaped bundle sheath cells, about 37.28 µm in diameter and showing additional thickening of cell walls (Figure 7A,B). Within the vascular bundles, sclerenchyma cells of *F. sacchari*-infected stems of corn plants were mostly polygonal in shape, with a mean diameter of 18.51 µm, (Figure 7B) and differed from those of uninfected corn stems by being distributed around the surroundings of the phloem and xylem vessels (Figure 7B) instead of being localized within the vascular cylinder (Figure 5B).

Furthermore, contrary to the predictable and organized distribution pattern of xylem and phloem vessels in stems of uninfected corn stems (Figure 5B), polygonal-shaped phloem vessels, about 6.59 µm in diameter, were surrounded randomly by numerous isodiametric xylem vessels, having a mean diameter of 29.01 µm, in corn stems infected with endophytic *F. sacchari* (Figure 7B). Additionally, unlike the vascular bundles of uninfected corn plants (Figure 5A,B), vascular bundles of *F. sacchari*-infected stems had a mean diameter of 98.90 µm and showed the absence of distinct protoxylem vessels (Figure 7A,B).

A significant decline in vascular bundle diameter, as well as cell sizes of cortical parenchyma, phloem, and metaxylem, was observed in stems of corn plants infected with endophytic *F. sacchari* (Figure 7A,B), compared to the uninfected control (Figure 5A,B) (*p* ≤ 0.05), whereas the diameters of bundle sheath cells of *F. sacchari*-infected corn stems (Figure 7B) were significantly larger than those of uninfected corn stems (Figure 5B) (*p* ≤ 0.05).

### 3.10. Histology of Corn Stems Infected with Endophytic P. citrinum

**Figure 8:**
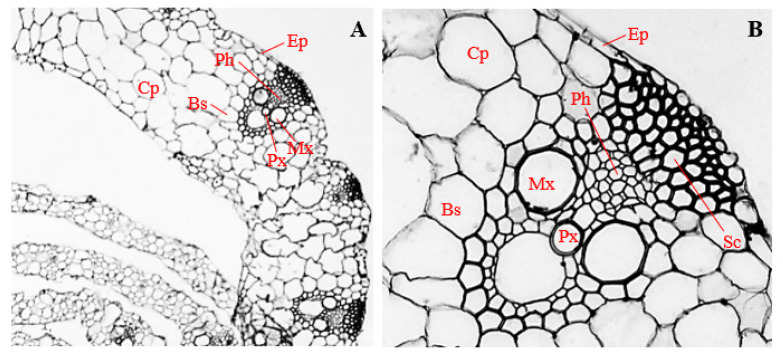
Transverse section of corn stem infected with endophytic *Penicillium citrinum*. (**A**) Magnification 200×; (**B**) magnification 400×. Ep, epidermis; Cp, cortical parenchyma; Sc, sclerenchyma; Ph, phloem; Mx, metaxylem; Px, protoxylem; and Bs, bundle sheath.

Stem cross-sections of corn plants infected with endophytic *P. citrinum* showed the presence of a uniseriate epidermis, which comprised mostly spindle-shaped to irregular cells, elongated horizontally, with a mean thickness of 7.26 µm (Figure 8A,B).

Isodiametric sclerenchyma cells with a mean diameter of 12.86 µm were observed immediately below the epidermal layer, at the upper end of the vascular bundle (Figure 8A,B). Within the vascular bundle, and slightly above the metaxylem vessels, polygonal phloem cells with a mean diameter of 9.21 µm were observed (Figure 8B). On opposite sides of the central region of the vascular cylinder, two polygonal to roughly circular metaxylem vessels were observed, with a mean diameter of 37.41 µm occurred (Figure 8A,B). In-between the metaxylem vessels, oval-shaped protoxylem vessels with a 29.83 µm mean diameter were observed (Figure 8B). The vascular cylinder, with a mean diameter of 146.16 µm, was encased by a bundle sheath made up of isodiametric cells and measuring about 34.89 µm in diameter (Figure 8A,B). The vascular bundle and bundle sheaths were surrounded by cortical parenchyma cells, shaped roughly circular to irregular and having a mean diameter of 57.81 µm (Figure 8A,B).

The thickness of the epidermis, and the diameter of the cortical parenchyma, phloem vessels, metaxylem vessels, sclerenchyma cells, and vascular bundle, were significantly reduced in stems of corn plants infected with endophytic *P. citrinum* (Figure 8A,B)*,* compared to the uninoculated control (*p* ≤ 0.05) (Figure 5A,B). However, stems of corn plants infected with endophytic *P. citrinum* had significantly larger diameters of bundle sheath cells (Figure 8A,B) compared to the uninfected control (*p* ≤ 0.05) (Figure 5B and Figure 8B).

### 3.11. Histology of Uninfected Corn Leaves

**Figure 9:**
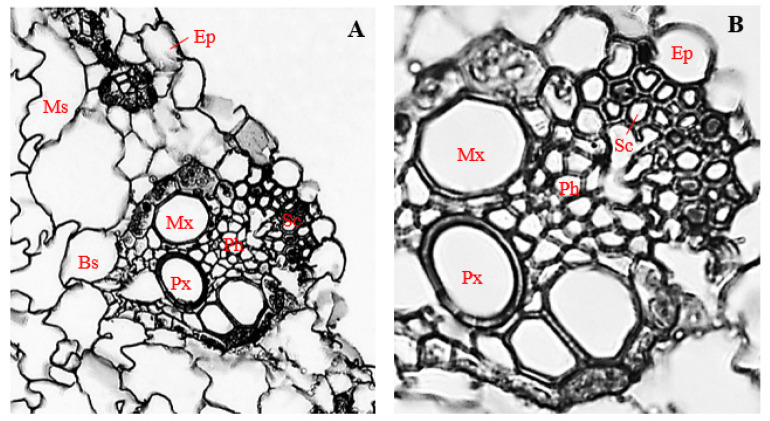
Transverse section of uninoculated corn leaf. (**A**) Magnification 200×; (**B**) magnification 400×. Ep, epidermis; Sc, sclerenchyma; Ph, phloem; Mx, metaxylem; Px, protoxylem; Bs, bundle sheath; and Ms, mesophyll.

Leaf cross-sections of uninfected corn plants (control) showed the presence of a uniseriately layered epidermis, with a mean thickness of 26.28 µm (Figure 9A,B). Epidermal cells were mostly oval, with a few shaped rectangularly and horizontally elongated (Figure 9A,B).

Immediately below the upper epidermis were the sclerenchyma cells. Cells of the sclerenchyma region were mostly polygonal, with a mean diameter of 10.70 µm (Figure 9A,B). Polygonal to rectangular-shaped phloem cells with a mean diameter of 10.17 µm were arranged below the sclerenchyma region, at an almost central position in the vascular bundle (Figure 9A,B).

Two to five isodiametric metaxylem vessels, measuring about 42.41 µm in diameter, were arranged on opposite sides of the vascular bundle, with an oval-shaped protoxylem about 35.89 µm in diameter in-between them (Figure 9A,B). The vascular bundles with mean diameter 155.88 µm were encased by bundle sheaths, made up of mostly irregularly shaped parenchyma cells, about 48.97 µm in diameter (Figure 9A,B). Densely packed and irregularly shaped mesophyll cells, about 67.16 µm diameter, were distributed throughout the leaf cortex and surrounded the vascular bundles (Figure 9A,B).

### 3.12. Histology of Corn Leaves Infected with Endophytic F. verticillioides

**Figure 10:**
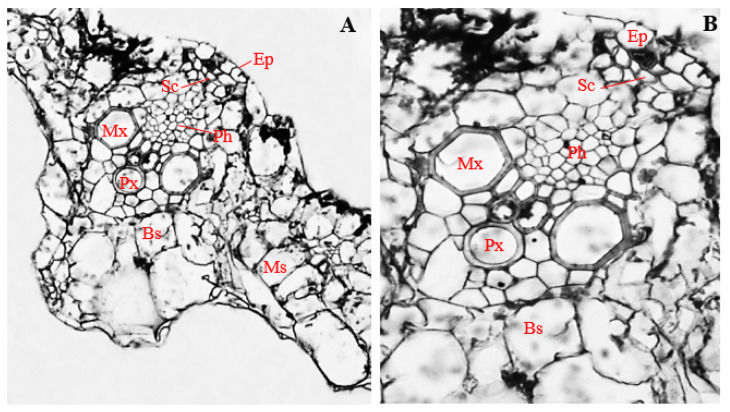
Transverse section of corn leaf infected with endophytic *Fusarium verticillioides*. (**A**) Magnification 200×; (**B**) magnification 400×. Ep, epidermis; Sc, sclerenchyma; Ph, phloem; Mx, metaxylem; Px, protoxylem; Bs, bundle sheath; and Ms, mesophyll.

Leaves of corn plants infected with endophytic *F. verticillioides* were sheathed by epidermal cells, about 8.56 µm thick (Figure 10A). Cells of the epidermal layer were uniseriate, horizontally elongated, and ranged in shape from roughly circular, to rectangular, and to irregular (Figure 10A,B). Beneath the epidermal layer, a few polygonal-shaped sclerenchyma cells, about 10.12 µm in diameter, were observed (Figure 10B).

In the vascular cylinder, polygonal phloem cells, 20.14 µm in mean diameter, were arranged slightly above the metaxylem vessels, towards the center of the vascular bundle (Figure 10B). Two to four metaxylem vessels, polygonal in shape, and with mean diameter of 25.57 µm, were positioned on opposite sides, at about the mid-point in the vascular cylinder (Figure 10B). Slightly below the center of the vascular cylinder, an oval-shaped protoxylem, about 20.14 µm in diameter, was situated (Figure 10A,B). The vascular bundle, about 118.27 µm in diameter, was enclosed by polygonal bundle sheath cells, with a mean diameter of 32.28 µm (Figure 10A,B). The bundle sheaths and vascular bundles were surrounded by large and irregularly shaped mesophyll cells, about 47.72 µm in diameter. Infected mesophyll cells showed proliferation of *F. verticillioides* mycelia (Figure 10A,B).

The thickness of the epidermis, and the diameters of phloem vessels, metaxylem vessels, protoxylem vessels, and vascular cylinder, were significantly reduced in leaves of corn plants infected with endophytic *F. verticillioides* (Figure 10A,B)*,* compared to the uninfected control (Figure 9A,B) (*p* ≤ 0.05).

### 3.13. Histology of Corn Leaves Infected with Endophytic P. citrinum

**Figure 11:**
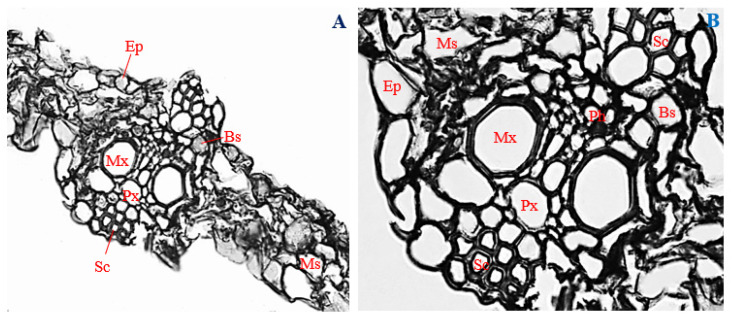
Transverse section of corn leaf infected with endophytic *Penicillium citrinum*. (**A**) Magnification 200×; (**B**) magnification 400×. Ep, epidermis; Sc, sclerenchyma; Ph, phloem; Mx, metaxylem; Px, protoxylem; Bs, bundle sheath; and Ms, mesophyll.

Leaves of corn plants infected with endophytic *P. citrinum* were sheathed in uniseriate epidermis, about 13.30 µm thick, and comprising horizontally elongated, rectangular to oval-shaped cells (Figure 11A,B).

Polygonal-shaped sclerenchyma cells, about 12.57 µm in mean diameter, were observed at both adaxial and abaxial positions of the vascular bundle of *P. citrinum*-infected leaves (Figure 11B), as opposed to leaf sections of uninfected plants, which had sclerenchyma cells at only the adaxial positions of the vascular bundle (Figure 9B).

Polygonal to roughly circular phloem vessels, about 6.47 µm in mean diameter, were observed slightly above the metaxylem vessels, towards the adaxial position of the vascular bundle (Figure 11B). Two isodiametric metaxylem vessels with mean diameter of 18.07 µm were found at opposite sides, around the central region of the vascular bundle (Figure 11B). Oval-shaped protoxylem vessels about 19.30 µm diameter were located slightly below the middle of the metaxylem vessels (Figure 11A,B). The vascular cylinder with mean diameter of 107.86 µm was enclosed by a bundle sheath which comprised irregular bundle sheath cells, with a mean diameter of 18.07 µm (Figure 11B). Vascular bundles and bundle sheaths were surrounded by irregular mesophyll cells, having mean diameter of 23.60 µm (Figure 11A,B).

Cells of the epidermis, cortical parenchyma, phloem, metaxylem, protoxylem, bundle sheath, and mesophyll of *P. citrinum*-infected leaves of corn plants (Figure 11A,B), were significantly reduced in size compared to the uninfected control (Figure 9A,B) (*p* ≤ 0.05), whereas leaves of corn plants infected with endophytic *P. citrinum* showed significantly larger sclerenchyma cells, and a larger vascular cylinder (Figure 11A,B), compared to the uninfected control (Figure 9A,B) (*p* ≤ 0.05).

### 3.14. Colonization of Corn Tissues by Endophytic F. verticillioides

**Figure 12:**
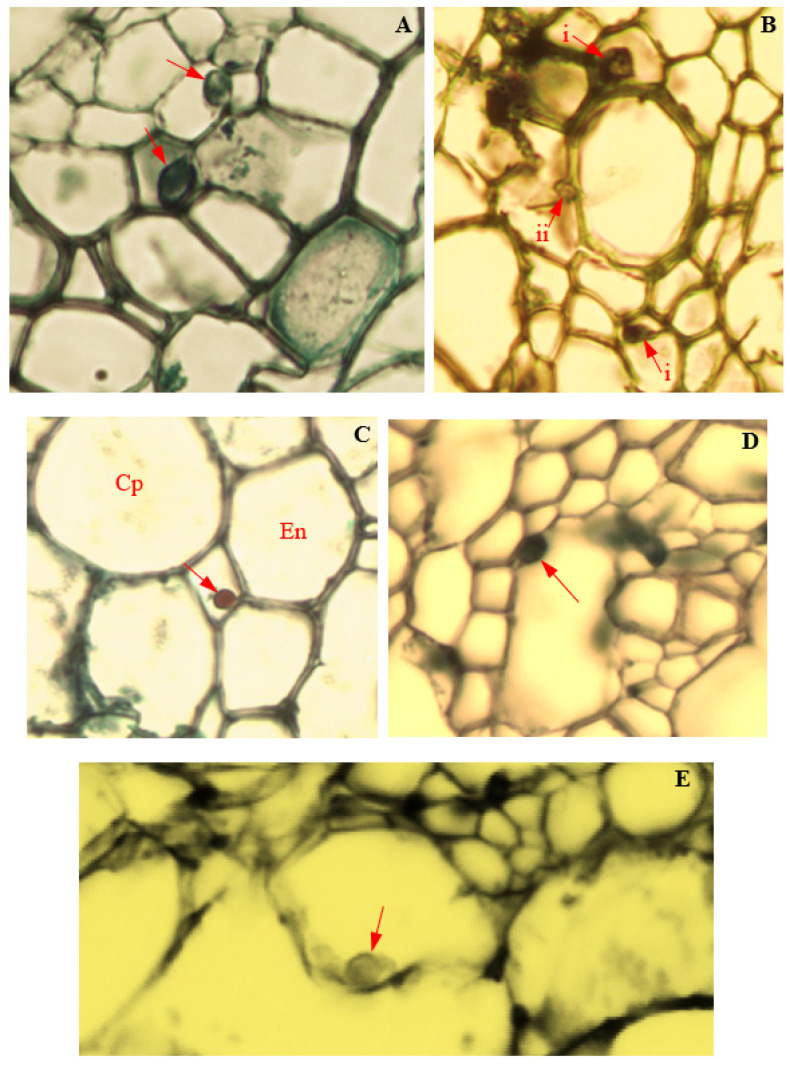
Light micrographs showing the endophytic colonization of corn tissues by *Fusarium verticillioides* (Magnification 400×). Red arrows indicate position of endophyte in the infected (**A**) root pericycle; (**B**) i: root pith; ii: root metaxylem; (**C**) intercellular cellular space between root cortical parenchyma (Cp) and endodermis (En); (**D**) stem protoxylem lacuna; and (**E**) leaf mesophyll.

**Figure 13:**
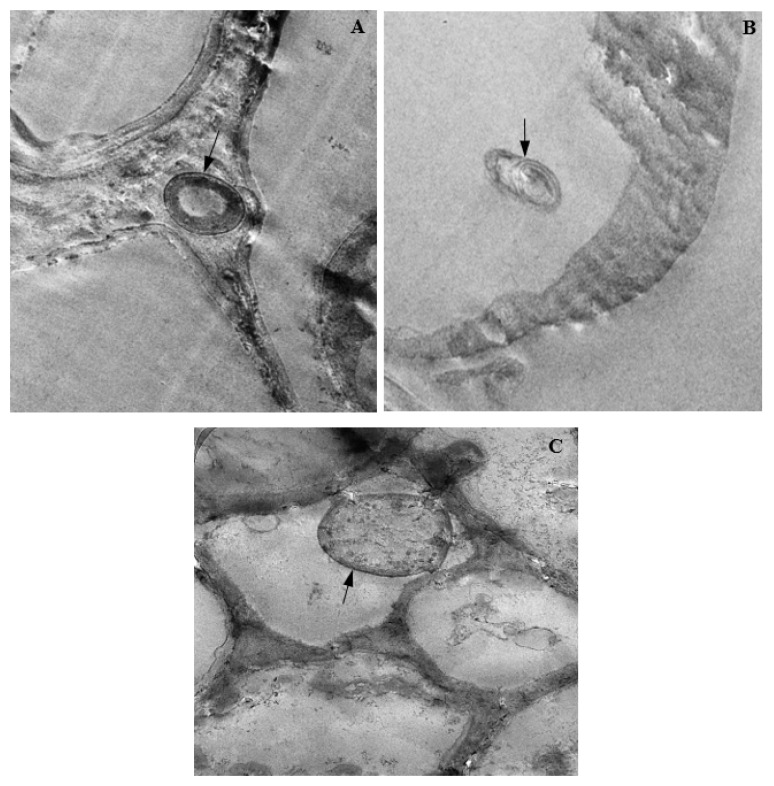
Transmission electron micrographs showing endophytic colonization of corn tissues by *Fusarium verticillioides*. The black arrows indicate the presence of hyphae in infected the (**A**) root intercellular space (Magnification 4000×); (**B**) stem parenchyma (Magnification 3200×); and (**C**) leaf mesophyll (Magnification 5000×).

Tissue colonization of corn plants by endophytic *F. verticillioides* was observed through LM and TEM (Figure 12 and Figure 13). In infected roots, the hyphae of endophytic *F. verticillioides* were attached to cell membranes of pericycle (Figure 12A), pith parenchyma, and protoxylem (Figure 12B). *Fusarium verticillioides* hyphae were also found attached to the wall of an endodermal cell, in the intercellular space between the cortical parenchyma and endodermis of infected root (Figure 12C). In the disintegrated vascular cylinder of infected corn stems, hyphal attachment of endophytic *F. verticillioides* to the wall of a remnant xylem parenchyma was also observed (Figure 12D). Intra-cellular colonization of infected corn leaves by endophytic *F. verticillioides* was evidenced by the presence of *F. verticillioides* hyphae in leaf mesophyll (Figure 12E). The observation of *F. verticillioides*-infected tissues of corn plants in TEM confirmed the colonization of root intercellular space (Figure 13A), stem parenchyma (Figure 13B), and leaf mesophyll cells (Figure 13C).

### 3.15. Colonization of Corn Tissues by Endophytic F. sacchari

**Figure 14:**
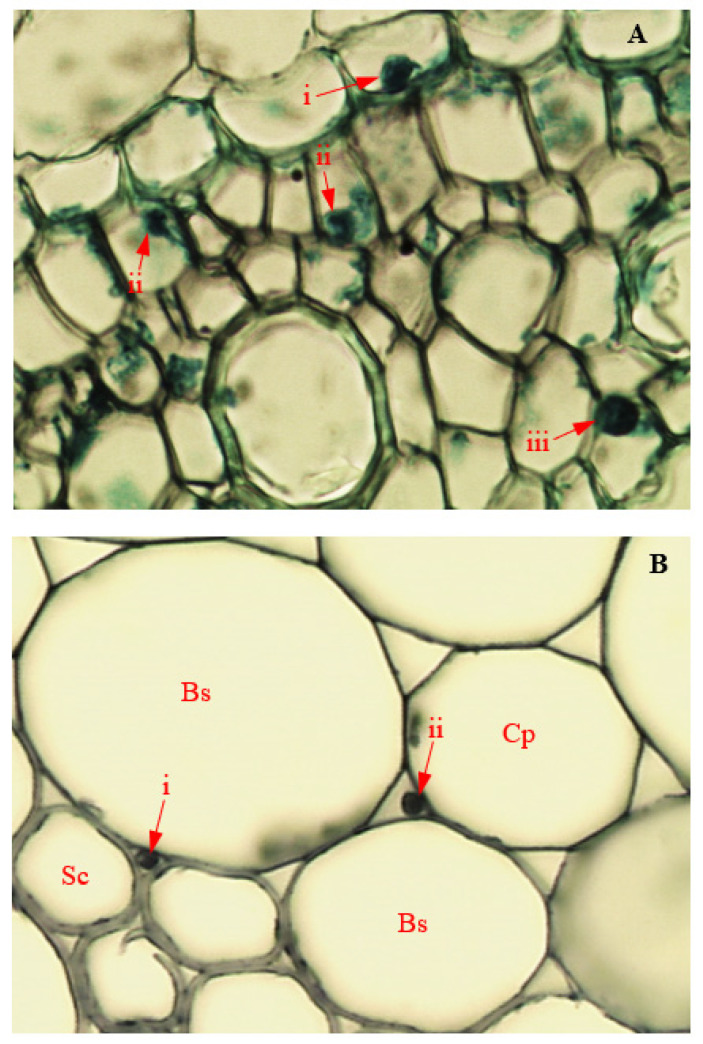
Light micrographs showing endophytic colonization of corn tissues by *Fusarium sacchari* (Magnification 400×). Red arrows indicate position of endophyte in infected (**A**) i: root endodermis; ii: root pericycle; and iii: root pith; (**B**) intercellular spaces between (**B**) i: stem sclerenchyma (Sc) and bundle sheath cells (Bs); and ii: stem bundle sheath cells (Bs) and cortical parenchyma (Cp).

**Figure 15:**
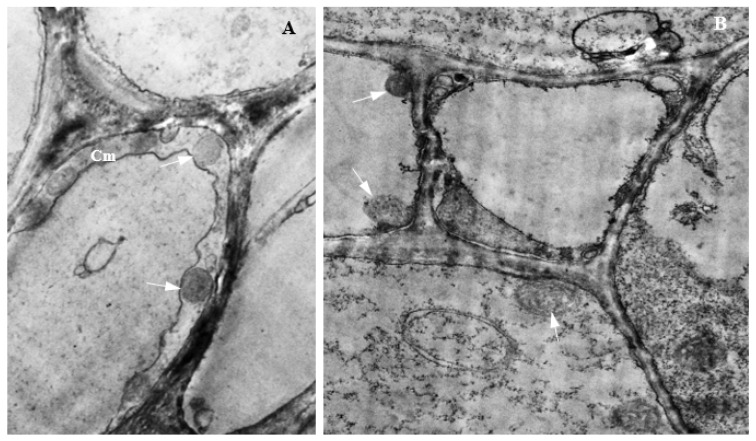
Transmission electron micrographs showing endophytic colonization of corn tissues by *Fusarium sacchari*. White arrows indicate the position of endophyte in (**A**) the presence of hyphae in cell membrane (Cm) of the infected root (Magnification 2500×); and (**B**) the attachment of hyphae to cell membranes of infected stem (Magnification 6300×).

Photomicrographs of corn tissue colonization by endophytic *F. sacchari* are presented in Figure 14 and Figure 15. In infected roots, hyphae of endophytic *F. sacchari* were seen attached to the membranes of the endodermis, pericycle, and pith cells (Figure 14A). In tissues of corn stems, intercellular colonization by endophytic *F. sacchari* was evidenced by the presence of *F. sacchari* hyphae in the intercellular spaces between the sclerenchyma cells and bundle sheath cells, and between the bundle sheath cells and cells of the cortical parenchyma (Figure 14B). The transmission electron microscopy of *F. sacchari*-infected corn tissues confirmed hyphal presence in the cell membranes of roots (Figure 15A), and attachment of *F. sacchari* hyphae to the cell membranes of infected stems (Figure 15B).

### 3.16. Colonization of Corn Tissues by Endophytic P. citrinum

**Figure 16:**
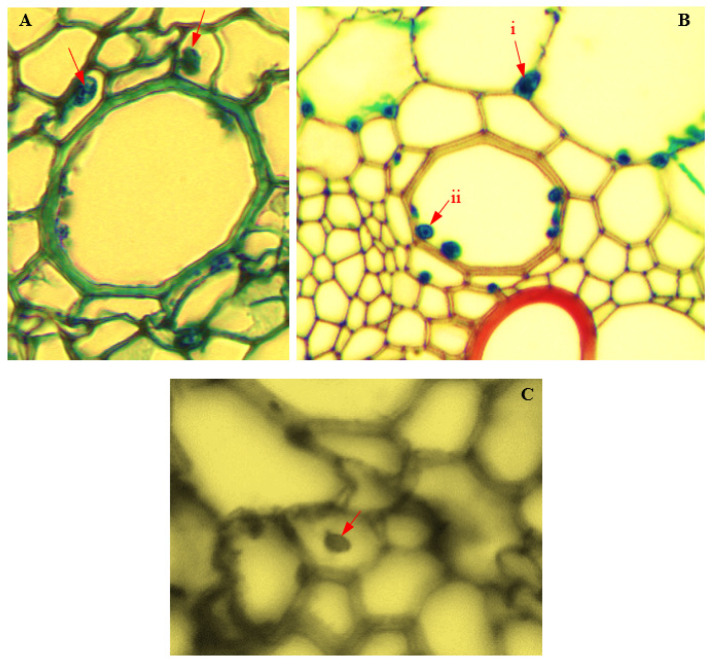
Light micrographs showing endophytic colonization of corn tissues by *Penicillium citrinum* (Magnification 400×). Red arrows indicate the position of endophyte in infected cells of (**A**) root pith; (**B**) i: stem bundle sheath; ii: stem metaxylem; and (**C**) leaf phloem.

**Figure 17:**
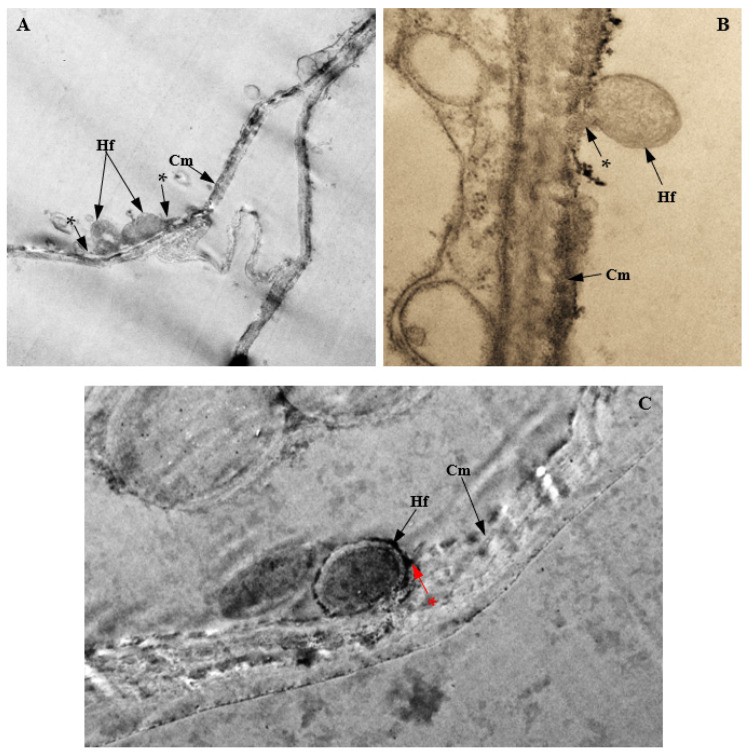
Transmission electron micrographs showing endophytic colonization of corn tissues by *Penicillium citrinum*. The attachment of hyphae (Hf) to cell membranes (Cm) of infected (**A**) root (Magnification 2500×); (**B**) stem (Magnification 12500×); and (**C**) leaf (Magnification 2000×). The penetration peg is marked with an asterisk (*).

Light microscopy and the TEM of *P. citrinum*-infected cells of corn plants showed the intracellular colonization of infected cells of corn plants (Figure 16 and Figure 17). In infected roots, intracellular attachment of *P. citrinum* mycelia to cell membranes of the parenchyma cells surrounding the protoxylem was observed (Figure 16A). In stem tissues, mycelia of endophytic *P. citrinum* were attached to walls of the bundle sheath cells and metaxylem vessels (Figure 16B), while leaf colonization by endophytic *P. citrinum* was evidenced by the presence of *P. citrinum* mycelia in the lumen of phloem cells (Figure 16C). Transmission electron microscopy of infected tissues showed the attachment of *P. citrinum* hyphae to cell membranes of colonized roots (Figure 17A), stems (Figure 17B), and leaves (Figure 17C).

## 4. Discussion

Endophytic colonization of corn plants in the present study led to significant alterations in the histological characteristics of corn plants. The infection of roots, stems, and leaves of corn plants by endophytic *P. citrinum*, *F. sacchari*, and *F. verticillioides* led to a significant decline in the diameter of phloem and metaxylem vessels. A reduction in the cell diameter of conducting tissues has been described as a defense mechanism by infected plants, to contain the spread of pathogens to other plant parts. Evidence provided by Pouzoulet et al. [23] showed that plants with wider diameters of xylem vessels were more susceptible to diseases caused by vascular pathogens, compared to those with narrower xylem diameters. It can thus be concluded that tissue colonization of corn plants by the endophytic fungi was able to trigger the activation of a vascular defense mechanism in the infected plants.

Corn plants infected with endophytic *F. verticillioides* showed occlusion of the root and stem xylem vessels, and degradation of the epidermis, cortex, and vascular cylinders of root and stem tissues. This was accompanied by a significant decline in the thickness of leaf epidermal layers, as well as cell diameters of the cortical parenchyma and the vascular bundle. Works on the histopathology of endophytic *F. verticillioides* on corn plants are currently unavailable. Lawrence et al. [6] reported the collapse of epidermal cells, cortical parenchyma, and vascular cylinder, accompanied by occlusion of protoxylem vessels in roots and stems of corn plants infected by a pathogenic strain of *F. verticillioides.* Muimba-Kankolongo [24] also stated that the shredding of the pith, accompanied by the discolouration of surrounding tissues, is significant in the final stages of corn tissue infection by *F. verticillioides.* Although vessel occlusion with tyloses and gels by infected plants is considered a protective mechanism targeted at preventing the distribution of pathogens to different plant parts through the conducting channels, it often leads to the blockage of conducting vessels, which is a major cause of vascular wilts in plants [25]. The degradation of infected tissues by endophytic *F. verticillioides* in this report could be a result of the activity of cell wall degrading enzymes synthesized by the fungus to facilitate tissue invasion and colonization. A gene coding for endopolygalacturonase, a cell-wall-degrading enzyme, was cloned from *F. verticillioides* by Caprari et al. [26]. The significant tissue colonization and disruption of vascular, cortical, and epidermal corn tissues by endophytic *F. verticillioides* in this report suggests that endophytic *F. verticillioides* are also capable of pathogenic behavior.

The infection of corn plants by endophytic *F. sacchari* led to significant thickening of the root epidermis, and both thickening and size increases of bundle sheath and sclerenchyma cells of infected stems. Although this is the first report of the histopathology of *F. sacchari* on any plant, similar cell responses have been reported in plant cells infected by other *Fusarium* species. For instance, Yates et al. [3] reported the accelerated deposition of lignin in shoot cells of corn plants infected by *F. verticillioides*. Cell wall reinforcement is an active component of the cell wall integrity (CWI) maintenance mechanism, which regulates CWI in response to external stimuli, including fungal infections [27]. Stress lignins are usually deposited as reinforcements of cell walls against further invasion by fungi and other infectious agents [28]. According to Ride [29], cell wall reinforcement makes cells resistant to mechanical penetration, provides resistance to wall dissolution by fungal enzymes, and avails phenolic precursors of lignin and free radicals, for the inactivation of fungal membranes, toxins, enzymes, and elicitors. In the present study, increase in sizes and thickness of bundle sheaths and sclerenchyma cells of corn plants infected by endophytic *F. sacchari* could be seen as reinforcements aimed at protecting plant tissues from further invasion by the endophyte. Similarly, Attia et al. [30] reported that a sharp decline in bundle sheath permeability in response to the presence of apoplastic chitin was able to confer chitin-triggered immunity, which is important for the prevention of cell infection by fungal pathogens. This also explains the confinement of endophytic *F. sacchari* within the intercellular spaces of infected corn roots in the present study. Li et al. [31] also reported that thickening of sclerenchyma cells near the epidermis inhibited penetration of rice leaves by *M. oryzae* at the early stages of infection.

Infection of corn plants by endophytic *P. citrinum* showed mild histopathological responses in host tissues. A significant increase in the sizes of root vascular bundle diameter, stem bundle sheath diameter, and diameter of leaf sclerenchyma was observed in *Penicillium citrinum*-infected corn plants. This is the first report of the histopathology of *P. citrinum* on any plant. Endophytic *P. citrinum* is a known producer of the growth promoter, gibberellic acid [32]. Plant growth improvement by *P. citrinum* has also been linked to the production of functional biochemicals, and the modification of antioxidant activities in plant tissues [33,34]. It is therefore possible that the increase in cell sizes of *P. citrinum*-infected plant tissues could be a result of enhanced hydraulic conductance, cell wall extensibility, turgidity pressure, and water potential difference, among the cells and their surroundings [32].

The initiation and development of disease in plant tissues by the endophytic *F. verticillioides, F. sacchari,* and *P. citrinum* could have also been influenced by factors such as age of plant, inoculum size, and method of inoculum application. Older and fully mature plants are generally more resistant to fungal infections compared to younger plants, as a result of the presence of more developed and advanced anatomical and physiological defense mechanisms in older plants compared to younger ones [35]. In corn plants, alleles at the *hm1* locus are able to sufficiently confer adult plant resistance (APR) at maturity but are weak and unreliable in protecting younger plants within the first three weeks of age [36]. This suggests that pathogenicity tests conducted on corn plants older than those used in the present study could reveal a disease response that may vary from the findings of this study. Disease severity is also generally reported to be directly proportional to inoculum size, with the highest disease severities attained at higher inoculum concentrations, and vice-versa in most plant–pathogen interactions [37]. It is thus likely that lower disease severities could have been observed in corn plants artificially inoculated with lower concentrations of conidial suspensions of endophytic fungi than were used in the study, and vice-versa. Furthermore, since the method of inoculation of corn plants is regarded as an important determinant of the extent of tissue colonization and pathogenicity of fungal pathogens [38], it is possible that variable pathogenic interactions between endophytic fungi and corn hosts could be observed using inoculation techniques different from those used in the present study.

## 5. Conclusions

The present study revealed that *F. verticillioides, F. sacchari,* and *P. citrinum* living endophytically in corn plants are capable of pathogenic behavior, with endophytic *F. verticillioides* being the most virulent. The intra- and inter-cellular colonization of host tissues by the endophytic fungi induced a significant reduction in the diameters of vascular, cortical, and epidermal tissues, accompanied by cell proliferation and degradation. Follow-up studies to consider the effect of varied conidial doses of endophytic fungi applied on host plants at different levels of maturity using different inoculation techniques, and the possible roles of pathogenicity-related compounds synthesized by the studied fungi in host tissues, shall provide a greater understanding of the extent and implication of host-endophyte relationship on the overall health and productivity of infected corn plants.

## Figures and Tables

**Table 1:** The percentage occurrence of endophytic *P. citrinum*, *F. verticillioides*, and *F. sacchari* in healthy husk and kernel tissues of corn plants.

Fungal Endophyte	Occurrence (%)	
	Husk	Kernel
*P. citrinum*	1.40	28.20
*F. verticillioides*	8.57	3.93
*F. sacchari*	8.57	0.00

**Table 2:** The disease symptoms initiated by endophytic fungi in infected corn plants.

	Disease Symptoms		
Endophyte	Roots	Stems	Leaves
*F. verticillioides*	Reduced root formation	Reduced stem elongation, stem narrowing, and stem rot	Leaf chlorosis and necrosis
*F. sacchari*	Reduced root formation	Stem narrowing, excessive stem elongation, and stem malformation	-
*P. citrinum*	Reduced root formation	Stem narrowing	Leaf chlorosis and necrosis

- = Fungi not recovered from plant part.

## Data Availability

The data presented in this study are openly available in FigShare at https://doi.org/10.6084/m9.figshare.16822879 (accessed on 17 October 2021).
